# Specific anti-SARS-CoV-2 S1 IgY-scFv is a promising tool for recognition of the virus

**DOI:** 10.1186/s13568-022-01355-4

**Published:** 2022-02-12

**Authors:** Shikun Ge, Rao Wu, Tingting Zhou, Xiang Liu, Jin Zhu, Xiaoying Zhang

**Affiliations:** 1 School of Biological Science and Engineering, Shaanxi University of Technology, Hanzhong, China; 2 Centre of Molecular and Environmental Biology, Department of Biology, University of Minho, Campus de Gualtar, 4710-057 Braga, Portugal; 3 Department of Biomedical Sciences, Ontario Veterinary College, University of Guelph, Guelph, ON Canada; 4 Huadong Medical Institute of Biotechniques, Nanjing, Jiangsu China

**Keywords:** Coronavirus disease 2019 (COVID-19), Severe acute respiratory syndrome coronavirus 2 (SARS-CoV-2), Spike (S) protein S1 subunit, Egg yolk antibody (IgY), Chicken IgY single chain variable antibody fragment (IgY-scFv), IgY technology

## Abstract

As severe acute respiratory syndrome coronavirus 2 (SARS-CoV-2) continues to spread globally, a series of vaccines, antibodies and drugs have been developed to combat coronavirus disease 2019 (COVID-19). High specific antibodies are powerful tool for the development of immunoassay and providing passive immunotherapy against SARS-CoV-2 and expected with large scale production. SARS-CoV-2 S1 protein was expressed in *E. coli* BL21 and purified by immobilized metal affinity chromatography, as antigen used to immunize hens, the specific IgY antibodies were extracted form egg yolk by PEG-6000 precipitation, and the titer of anti-S1 IgY antibody reached 1:10,000. IgY single chain variable fragment antibody (IgY-scFv) was generated by using phage display technology and the IgY-scFv showed high binding sensitivity and capacity to S1 protein of SARS-CoV-2, and the minimum detectable antigen S1 protein concentration was 6 ng/µL. The docking study showed that the multiple epitopes on the IgY-scFv interacted with multiple residues on SARS-CoV-2 S1 RBD to form hydrogen bonds. This preliminary study suggests that IgY and IgY-scFv are suitable candidates for the development of immunoassay and passive immunotherapy for COVID-19 to humans and animals.

## Introduction

COVID-19, first reported in December 2019, can cause pneumonia-like symptoms, including fever, cough and fatigue. There are no specific drugs available for COVID-19 at present (Le Bert et al. 2020; Long et al. 2020). The pathogen, SARS-CoV-2, is positive-sense single-stranded RNA virus with a genome of approximately 30 kbp encodes four structural proteins, including spike, envelop, matrix and nucleocapsid (Kim et al. 2021). The spike (S) protein comprises two components: S1, which contains the distinct receptor binding domain (RBD); S2, which contains the fusion peptide. The viruses enter the host cells through the interaction of the RBD with host cell receptor angiotensin converting enzyme 2 (ACE2) (Wang et al. 2020). Antibodies against the S1 subunit, particularly targeting to RBD, are expected to prevent the viral particle surface protein from binding to the ACE2 receptor, thus preventing viral invasion (Parray et al. 2020). Therefore, the SARS-CoV-2 S1 protein has been accepted as ideal target for antibody and vaccine design (Premkumar et al. 2020; Parray et al. 2020). Furthermore, antibodies are the powerful tool for the development of point-of-care testing (POCT) immunoassay in SARS-CoV-2 analysis, the desired method should be accurate, specific, rapid, robust and inexpensive (Seo et al. 2020; Kim et al. 2021).

Apart from the routinely used IgG, IgY is the evolutionary precursor of IgG, avian sourced IgY antibodies can be also served as the counterpart of IgG, and the polyclonal IgY can be easily obtained in large quantities from hen egg yolk for broad biomedical purposes owning to a series of advantages such as higher productivity, better animal welfare, higher immunogenicity to mammal conserved proteins, lower cross-reactivity and not react with rheumatoid factors to avoid false positive results in immunoassay, as compared to the generation and application of mammalian full length monoclonal antibody (mAb) (Schade et al. 2007; Ge et al. 2020). Furthermore, in the recent decades, monoclonal IgY, particularly the IgY-scFv can be generated by using the phage display technology to better combine the biological superiorities of both IgY and recombinant antibody fragment (smaller size), is gaining increasing attention and application (Lee et al. 2017). The chicken monoclonal IgY-scFv has a high binding capacity to targeted pathogens (Chi et al. 2002; Wang et al. 2006; Pavoni et al. 2006), and it can overcome the difficulties in generating highly specific monoclonal antibodies (mAbs) from the traditional way of antibody generation, such as advantages of low cost and high yield (Groves and Morris 2000). Our previous studies demonstrated that IgY-scFv can be generated and applied in different immunoassays for the detection of small molecules gentamicin (Li et al. 2016) and large molecules CPV-VP2 (Ge et al. 2020) in the veterinary practice. It has been reported that scFv antibodies generated by using phage display technology has high neutralizing effect against SARS-CoV infection (Sui et al. 2004; Kang et al. 2006).

IgY have been extensively studied for the treatment of various respiratory viruses prior to the COVID-19 (Xiao et al. 2019), and IgY have also made some achievements in the treatment of pathogenic coronaviruses (Park and Iwasaki 2020), as early as in 2003 after the SARS epidemic, there was a research on IgY against SARS-CoV (Fu et al. 2006). In recent years, IgY antibodies have been developed and accessed targeting to coronavirus, the anti-MERS-CoV spike protein specific IgY have an efficient neutralization of virus infection in both in vitro and in vivo (El-Kafrawy et al. 2021), the specific IgY from hens immunized with inactivated SARS-CoV-2 had significant inhibitory against in vitro infections with live and pseudo typed SARS-CoV-2, and could stay in the upper airways for hours administered via oral spray or nasal drip (Shen et al. 2021).

This study aimed to generate and evaluate the IgY-scFv against SARS-CoV-2 S1 protein, which may offer an alternative solution in providing large number of highly-specific antibodies in combating COVID-19 in different application scenarios.

## Materials and methods

### Preparation of SARS-CoV-2 S1 protein

The full-length SARS-CoV-2 S1 gene (GenBank: QHD43416.1) was synthesized by Genscript Inc. (Nanjing, Jiangsu, China), and inserted between *Bam*H I and *Not* I restriction sites in a pET-28a (+) plasmid (Novagen, Beijing, China), transformed into *E. coli* (BL21, DE3) and induced overnight at room temperature using isopropyl-β-d-thiogalactopyranoside (IPTG, 0.1 mM). Cells were collected by centrifugation and resuspended in phosphate buffered solution (PBS, 0.01 M, pH 7.4). After sonicated, the precipitates (inclusion bodies) were dissolved in PBS (containing 8 M urea) and dialyzed against Tris–HCl (20 mM, pH 7.4, containing sequential dilutions of urea). The recombinant S1-6 × His protein was purified by immobilized metal affinity chromatography. Protein concentration was determined by OD absorbance at 280 nm.

### Immunization of hens

Three twelve-week-old white Leghorn hens were immunized intramuscularly with SARS-CoV-2 S1 protein mixed with Freund’s adjuvant (Sigma-Aldrich, St. Louis, MO, USA) at five different sites of breast muscles. S1 protein (250 µL, 1 mg/mL) in equal volume of normal saline was emulsified with Freund’s complete adjuvant (FCA; Sigma-Aldrich, St. Louis, MO, USA) in the first immunization, and four booster immunizations were followed up with Freund’s incomplete adjuvant (FIA; Sigma-Aldrich, St. Louis, MO, USA) at 2-week intervals in the breast muscles. All eggs were collected from the first immunization and were stored at 4 °C for future use. All experimental animal protocols were reviewed and approved by the institutional Ethics Committee for the use of laboratory animals.

### Generation of polyclonal IgY

IgY antibody was extracted from egg yolk by polyethylene glycol (PEG) precipitation. Twice the egg yolk volume of PBS was mixed with the yolk, PEG-6000 (3.5% w/v) were added to mixture and vortexed to remove lipids and lipoprotein, followed by 30 min rolling on a rolling mixer, and the mixture was centrifuged at 10,000*g* 4 °C for 20 min. The supernatant was poured through a filter and transferred to a new tube and PEG-6000 (8.5% w/v) was added to the tube, vortexed and rolled on a rolling mixer for 30 min. The mixture was centrifuged to remove supernatant and the precipitation was carefully dissolved in 10 mL PBS, PEG-6000 (12% w/v) was added into the tube on a rolling mixer for 30 min, and the mixture was centrifuged, the precipitation was dissolved in 1.2 mL PBS and the mixture was dialyzed 24 h in PBS at 4 °C. The IgY antibody extract was harvested from the dialysis capsule and transferred to 2 mL tube, stored at − 20 °C for future use. SDS-PAGE and Western blot were conducted to evaluate the purity and specificity of the obtained IgY, and ELISA was performed to analyze antibody titer.

### Assembly of IgY-scFv

The spleen tissue from the hen with the highest IgY titer was collected to extract the total RNA by using Total RNA Kit (Tiangen Biotech, Beijing, China), and the first-strand cDNA was synthesized by HiScript Q Select RT SuperMix for PCR (Vazyme Biotech, Nanjing, China). The heavy variable fragment (V_H_) and light chain variable fragment (V_L_) genes were amplified by PCR with primers HF-*Sfi* I and HR-Linker, LF-Linker and LR-*Not* I, respectively (Table 1). The V_H_ and V_L_ which both contained the sequence of a peptide linker were assembled to scFv with primers HF-*Sfi* I and LR-*Not* I by Overlap PCR.

**Table 1:** Primers used for PCR

Name	Primer sequences (5ʹ–3ʹ)	Application
HF-*Sfi* I	ATGTCTATGGCCCAGCCGGCCGTGACGTTGGACG	V_H_
HR-Linker	CAGAGCCACCTCCGCCTGAACCGCCTCCACCGGAGGAGACGATGACTTCGG	V_H_
LF-Linker	TTCAGGCGGAGGTGGCTCTGGCGGTGGCGGATCGGCGCTGACTCAGCCGTCCT	V_L_
LR-*Not* I	AGTTACTGGAGCGGCCGCACCTAGGACGGTCAGGG	V_L_

### Construction of IgY-scFv antibody library

The scFv gene products were digested with *Sfi* I and *Not* I restriction enzyme and ligated to pCANTAB5E vector (Thermo Fisher Scientific, Waltham, MA, USA) by T4 DNA Ligase (NEB, Ipswich, MA, USA). The ligated products were transformed into *E. coli* TG1 cells and plated onto SOBAG plates (containing 2% tryptone, 0.5% yeast extract, 0.05% NaCl, 2.5 mM KCl, 10 mM MgCl_2_, 1.5% agar powder, 100 μg/mL ampicillin and 2% glucose), the plates were incubated overnight at 37 °C to determine the scFv-TG1 library size and make the scFv-TG1 antibody library size reach more than 10^6^ pfu/mL.

### Bio-panning of IgY-scFv antibody library

Recombination phage library was generated in above scFv-TG1 library by the addition of helper phage *M13KO7* (NEB, Ipswich, MA, USA), precipitated with polyethylene glycol 8000 (4%, PEG-8000) and NaCl (3%, w/v), and resuspended in 2YT medium and stored at 4 °C. The bio-panning was carried out by adding 10^12^ plaque forming units (pfu) of recombinant phages to wells pre-coated with S1 protein followed by incubation at 37 °C for 2 h. The unbound phages were removed by Tween-PBS (PBST), bound phages were eluted with HCl-glycine (pH 2.5, 0.2 M) with end-over-end mixing for 10 min, the lower pH of the eluted phage neutralized with Tris–HCl (pH 9.0) and used to infect the TG1 *E. coli*. The amplified phages were precipitated and recovered as described above for the next round of selection, and the bio-panning was repeated for three more times. The enrichment of specificity was determined from the input/output ratio of the phage. Phage-infected TG1 cells from each bio-panning were plated onto SOBAG plates to determine the library size. Ten random scFv-phage clones on the plates were selected from the fourth round to identify the positive rate and binding capacity to antigen by PCR and phage ELISA, respectively.

### Phage ELISA

The binding capacity of scFv-phage suspensions to SARS-CoV-2 S1 protein were detected by phage ELISA. In detail, the bacteria solution (50 μL) from each antibody phage library was inoculated into 2YT medium (5 mL) and cultured at 37 °C for 3 h with shaking and transferred into of 2YT-AG medium (5 mL; containing 100 μg/mL ampicillin, 2% glucose) and *M13KO7* phages were added. The above 2YT-AG medium was incubated at 37 °C for 2.5 h at 150 rpm in the shaker and then centrifuged at 1000*g* for 15 min at 4 °C. The precipitate was resuspended in 2YT-AK (400 μL) and cultured at 37 °C overnight with shaking at 250 rpm on the shaker. The bacterial culture solution was centrifuged and the supernatant was collected for phage ELISA analysis.

The wells of 96-well Maxisorp microtiter plate (Nunc, Roskilde, Denmark) were coated with S1 protein in carbonate buffer solution (CBS, 100 μL) overnight at 4 °C and then blocked with bovine serum albumin (BSA, 300 μL) for 2 h at 37 °C. The scFv-phage was added to each well for 2 h incubation at 37 °C. The bound scFv-phage was detected with horseradish peroxidase (HRP)-conjugated anti-M13 antibody (Sino Biological, Beijing, China). The color was developed using TMB (Promega Biotech, Beijing, China) for 10 min and the absorbance was read at 450 nm.

### Expression and purification of anti-SARS-CoV-2 S1 IgY-scFv protein

The scFv with the highest binding capacity, as well as the corresponding pET-30a (+) vector, were digested by the *Bam*H I and *Hin*d III restriction enzyme (NEB, Ipswich, MA, USA), respectively, and the scFv was ligated into pET-30a (+) vector using the T4 DNA ligase (NEB, Ipswich, MA, USA). The positive recombinant plasmid was transformed into BL21 (DE3) and cultured in the LB medium (containing 1% tryptone, 0.5% yeast extract, 1% NaCl and 100 μg/mL kanamycin) at 37 °C. When the OD600 value reached 0.5–0.8, the scFv proteins were induced by IPTG (0.1 mM) for 8 h at 30 °C, the bacteria cells were collected by centrifuging at 12,000*g* for 10 min and resuspended in PBS. The mixture was sonicated (25% W, working 3 s and pause 3 s, total 15 min) and centrifuged at 10,000*g* for 10 min to collect the supernatant and sediment for SDS-PAGE analysis. The specificity of scFv was analyzed by Western blot.

### Analysis on IgY-scFv sensitivity by ELISA

A 96-well Maxisorp microtiter plate (Nunc, Roskilde, Denmark) was coated with different amounts of SARS-CoV-2 S1 protein (0.1, 0.2, 0.4, 0.6, 0.8, 1, 5, 8 and 10 ng/µL) dissolved in CBS, and incubated overnight at 4 °C, PBS was used as blank control (BC). The wells were rinsed with PBST (3 times × 5 min) and incubated with different amounts of scFv protein (0.2, 0.6, 1, 5 and 10 ng/µL) diluted in PBST for 1 h at 37 °C, PBS was used as negative control (NC). The wells were rinsed with PBST (3 times × 5 min), and the mouse anti-His monoclonal IgG conjugated with HRP were added (1:5000, 100 µL/well; CWBio, Beijing, China) and washed with PBST (3 times × 5 min). The TMB (Promega Biotech, Beijing, China) was added to each well for 20 min, and then the termination solution was added to read the absorbance at 450 nm.

### Molecular modeling and scFv-S1 protein docking study

Based on sequence identity/similarity homology, the SWISS-MODEL was used to build the molecular model of the scFv and RBD. Docking was performed by Discovery Studio 4.5 software (BIOVIA, San Diego, CA, USA), the scFv molecular model was docked with RBD using the ZDock Molecular Dock Program to obtain the docking conformation. The Residues Contact Frequency (RCF) algorithm was used to identify the most likely binding interface between scFv and RBD.

### Nucleotide data of the No.5 phage clone scFv

The No.5 phage clone scFv gene sequence has been uploaded to the GenBank database (GenBank accession number: OL743524).

## Results

### Evaluation of immunogen and polyclonal IgY

**Fig. 1:**
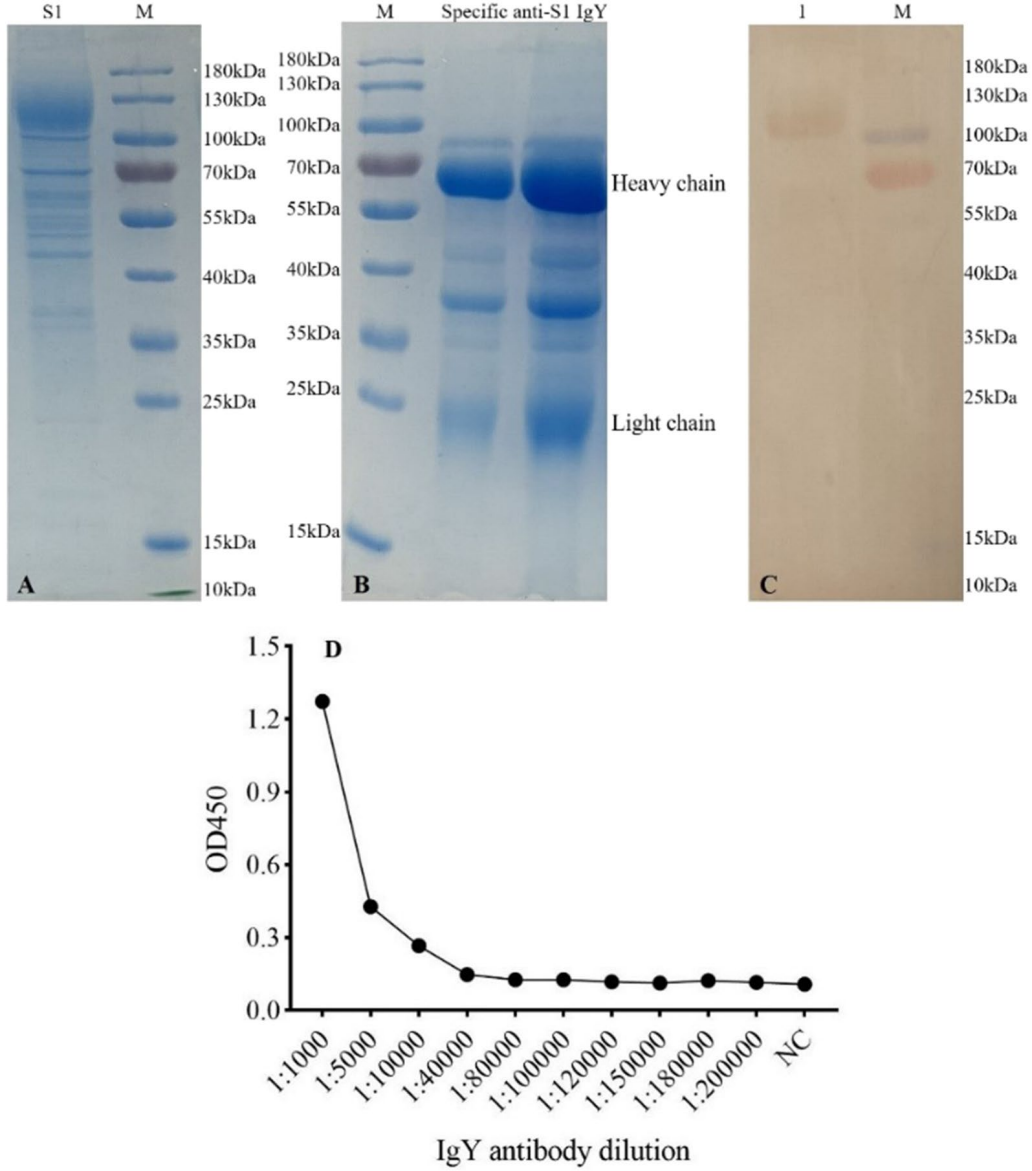
Characteristics of the S1proteien and specific anti-S1 IgY antibody. **A** SDS-PAGE analysis of the S1proteien. **B** SDS-PAGE analysis of the specific anti-S1 IgY antibody. **C** Western blot analysis of the specific IgY antibody. Lane 1, antigen was S1 protein, incubated with primary antibody IgY. **D** the analysis of the specific IgY antibody titer; Negative control sample (NC): PBS; Statistical principle, P/N > 2.0 (P, samples value; N, NC value), results are means of three replicates. M, Pre-stained Protein Ladder (Thermo Fisher Scientific, Waltham, MA, USA). The secondary antibody, goat anti-chicken IgY H&L (HRP) (Abcam, Cambridge, UK)

SARS-CoV-2 S1 protein was expressed in a prokaryotic expression system and characterized (Fig. 1A). The anti-S1 protein IgY was extracted and the SDS-PAGE gel indicated the light chain (23 kDa) and heavy chain (67 kDa) of IgY (Fig. 1B) (Pauly et al. 2009). The S1 protein could bind to specific IgY with a clear and specific single band (Fig. 1C). The specific IgY titer reached 1:10,000 (Fig. 1D).

### Construction of IgY-scFv library

**Fig. 2:**
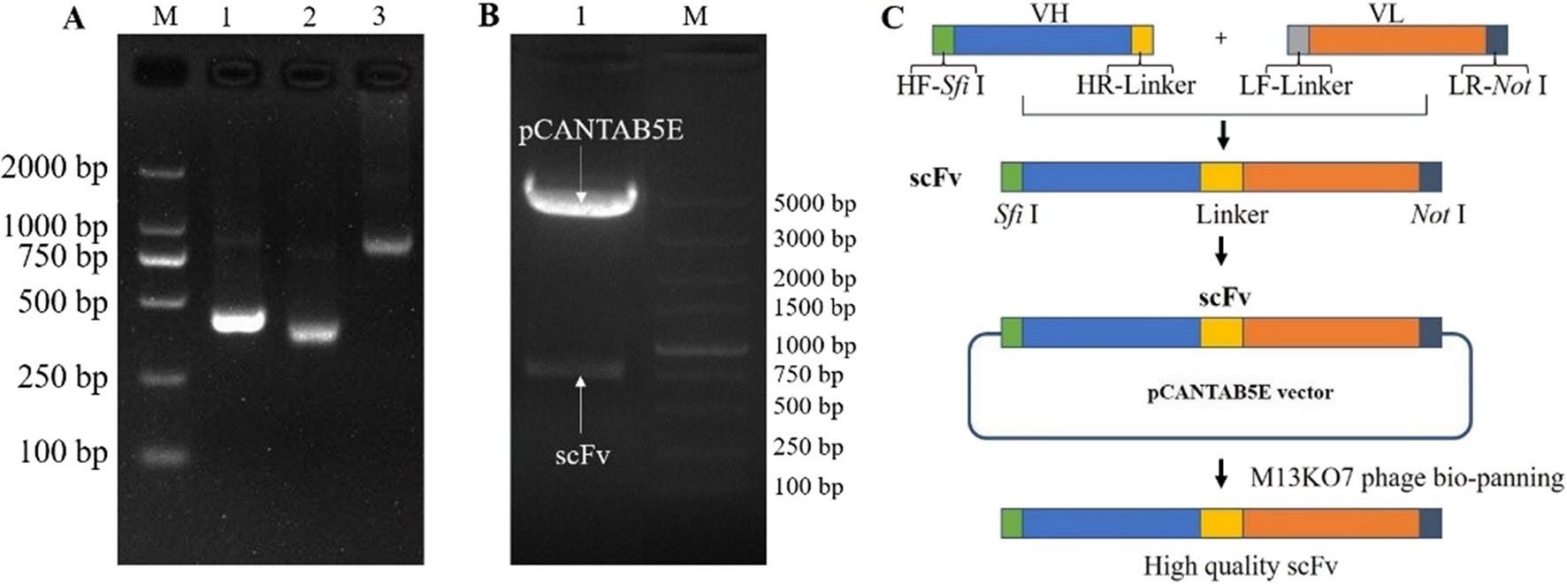
Construction of the IgY-scFv antibody phage display library. **A** PCR products of V_H_ (420 bp, lane 1), V_L_ (370 bp, lane 2) and scFv (800 bp, lane 3). **B** Double restriction digestion result of pCANTAB5E-scFv recombinant plasmid. **C** Schematic diagram of scFv construction. M: DNA marker (TaKaRa, Dalian, China)

The length of V_H_-linker, V_L_-linker and scFv were approximately 420 bp, 370 bp, and 800 bp, respectively (Fig. 2A and B). The titer of the primary anti-SARS-CoV-2 S1 scFv-TG1 library and the amplified *M13KO7* phage scFv library were 3.2 × 10^6^ pfu/mL and 2.8 × 10^13^ pfu/mL, respectively. Ninety-six percent of the phages contained the target fragments in the primary scFv-phage library.

### Bio-panning of IgY-scFv phage library

**Fig. 3:**
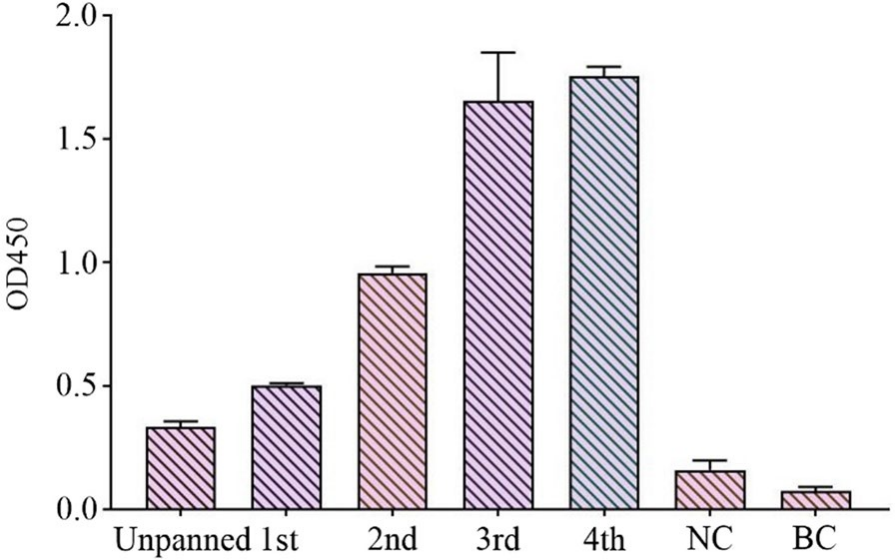
ELISA for 4 rounds of bio-panning phage library. Samples from round 1–4, phage in each of 4 rounds of bio-panning procedure. Unpanned sample: the primary scFv phage library. Negative control sample (NC): PBS. Blank control sample (BC): CBS

**Fig. 4:**
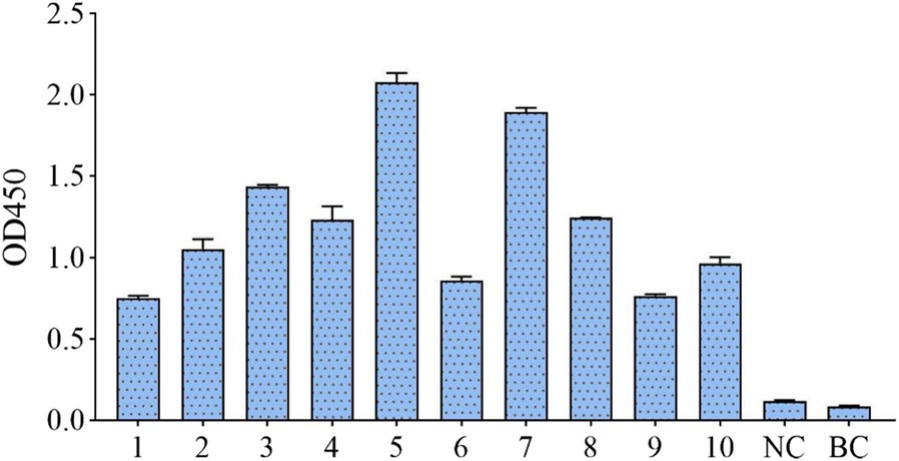
Reactivity of IgY-scFv to S1 protein analyzed by phage ELISA. 1–10, scFv antibodies from randomly selected clones from the fourth bio-panning. Negative control sample (NC), PBS. Blank control sample (BC), CBS

**Fig. 5:**
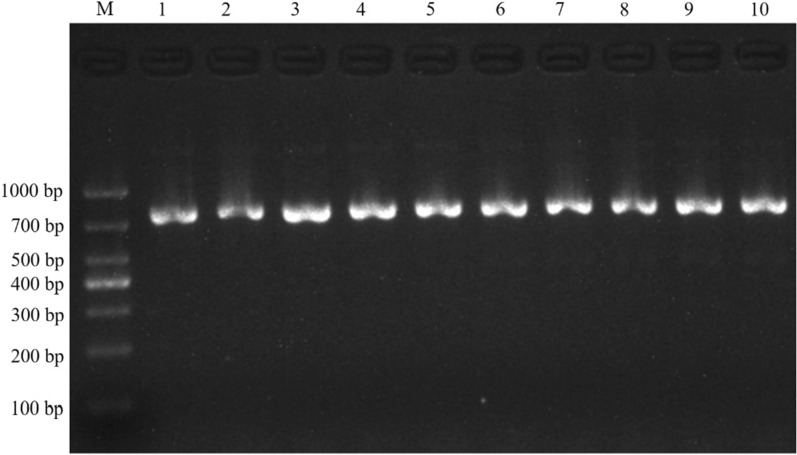
Identification of the ten IgY-scFv antibodies. 1–10, IgY-scFv from randomly selected clones at the fourth bio-panning. M, DL 1000 DNA marker

**Fig. 6:**
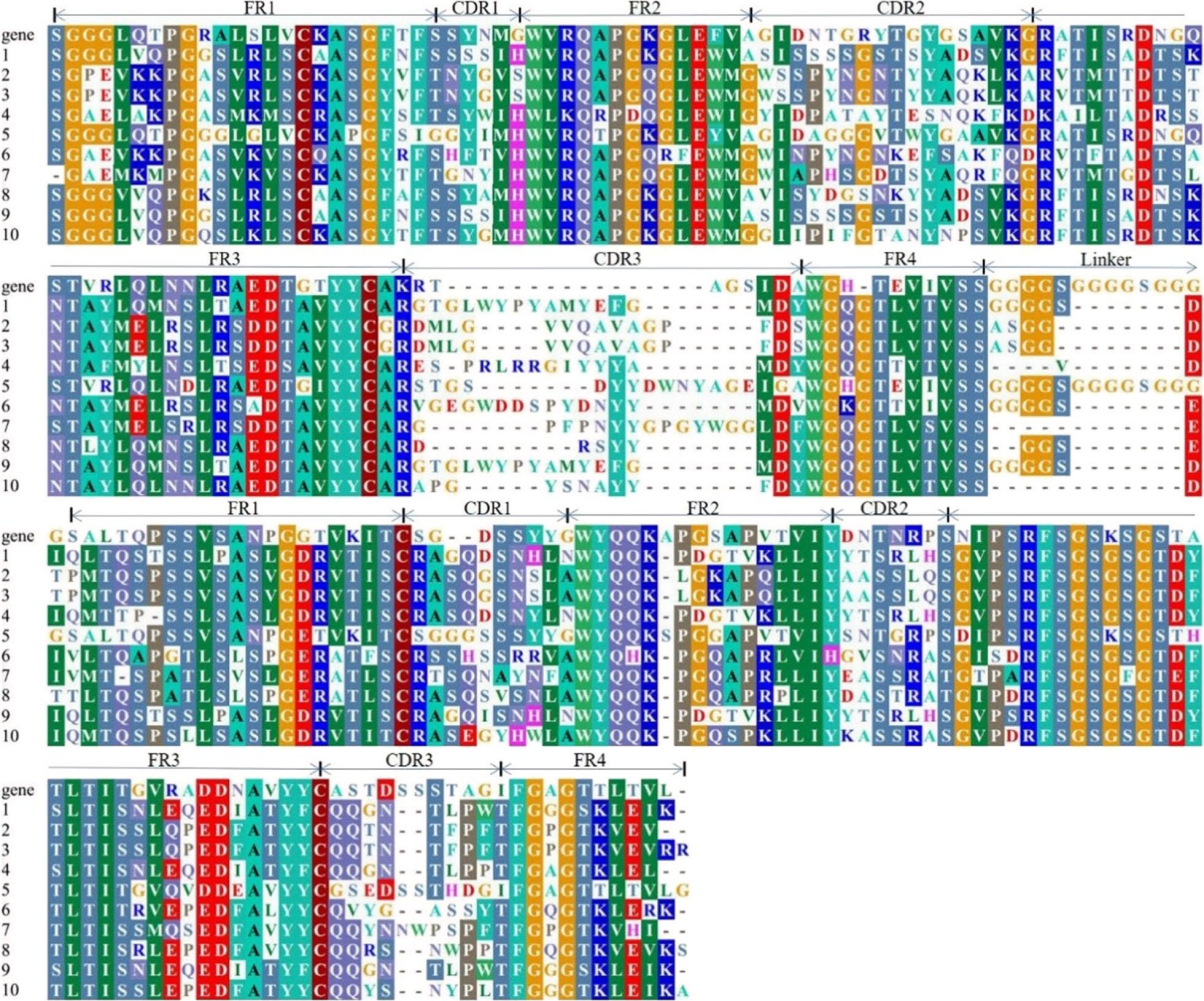
Sequence analysis of V_L_ and V_H_ genes of IgY-scFv. scFv antibody from randomly selected clones from the fourth bio-panning. The amino acid sequences of V_H_ and V_L_ in ten scFv clones to be aligned with those of the chicken germline sequence (gene) (Kovacs-Nolan and Mine 2004). FR: Framework region; CDR: complementarity determining region

The output of scFv-phage library was enriched 100 times in the fourth round as compared to the first round (Table 2), and the maximal ELISA binding signal was observed in the fourth round (Fig. 3). Ten randomly selected scFv-phage clones from the fourth-round bio-panning, in phage ELISA, each scFv-phage clone sample showed high binding activity against S1 protein, The No.5 scFv-phage showed the highest binding capacity and was chosen for further experiments (Fig. 4). All of the 10 clones contained expected scFv DNA sequences of about 800 bp (Fig. 5), and all the 10 scFv genes have complementary determining region 3 (CDR3) that was the main mutation region in both V_H_ and V_L_ (Fig. 6).

**Table 2:** Library sizes and phage titers of each bio-panning

Round	Coating concentration (per well)	Input (pfu/mL)	Output (pfu/mL)	Amplification library (pfu/mL)
1	15 µg	1 × 10^12^	5.6 × 10^6^	5.14 × 10^12^
2	10 µg	1 × 10^11^	7.2 × 10^6^	7.2 × 10^12^
3	10 µg	1 × 10^11^	2.8 × 10^8^	3.88 × 10^12^
4	5 µg	1 × 10^11^	6.6 × 10^8^	–

### Evaluation of polyclonal IgY-scFv

**Fig. 7:**
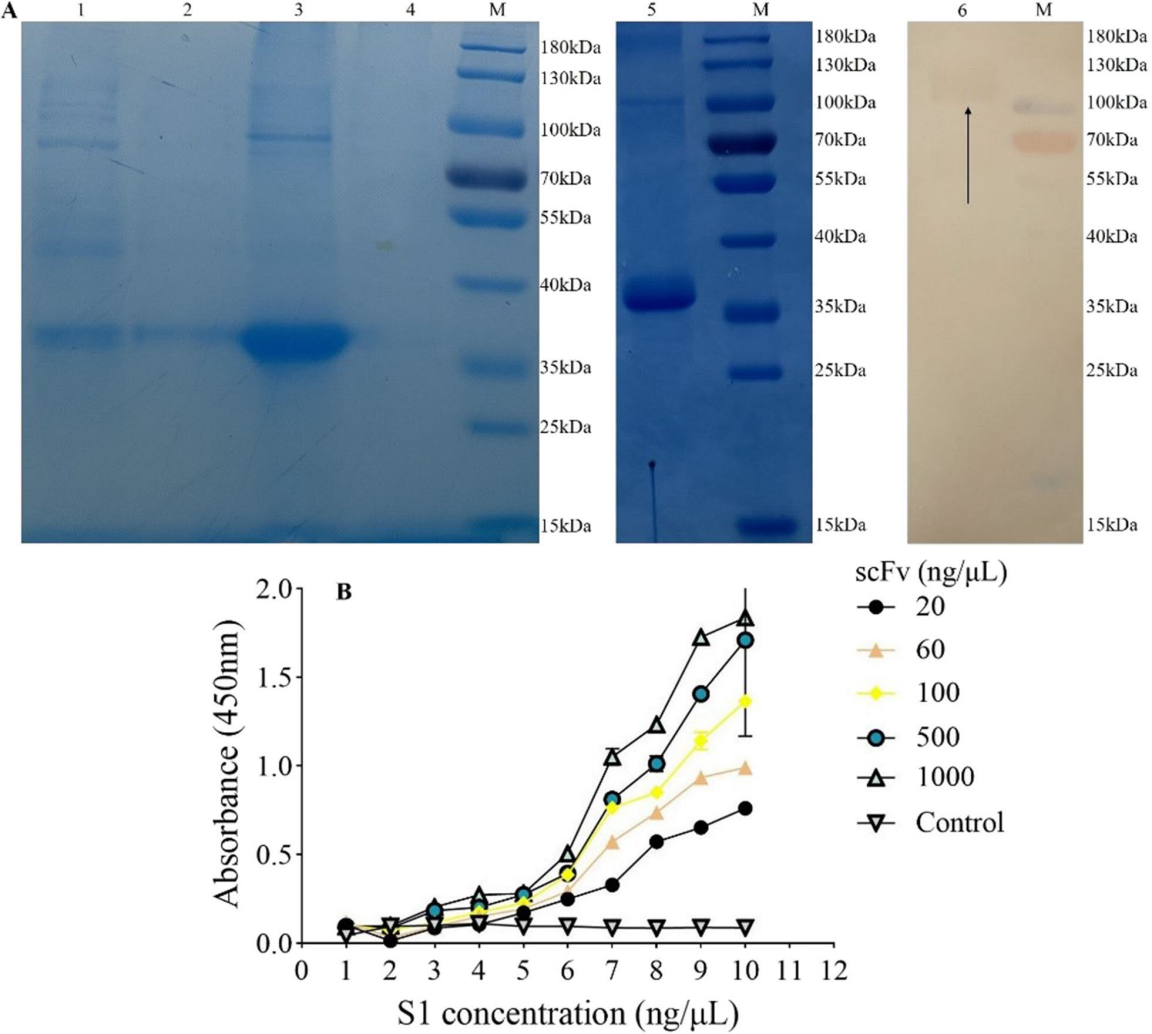
Characteristics of the IgY-scFv antibody. **A** ScFv expression, purification and western blot. Lanes 1 and 2: the pET-30a induced and non-induced. Lanes 3 and 4: the inclusion body and supernatant after cell disruption of scFv. Lanes 5: the denatured, renatured and purified scFv protein. Lanes 6: antigen was S1 protein, incubated with primary antibody scFv. M, Pre-stained Protein Ladder. **B** Analysis of IgY-scFv sensitivity. Control group, PBS; Statistical principle, P/N > 2.0 (P, samples value; N, control value), data of each point represented as Mean ± SD of triplicate

The scFv of No.5 phage clone was used for subsequent expression. The His-tagged scFv was overexpressed in an *E. coli* system, a band of estimated molecular mass of 38 kDa was detected on SDS-PAGE (Fig. 7A). The solubility analysis indicated that the expressed scFv protein existed as inclusion body (Fig. 7A), the denaturation and purification of scFv inclusion body protein showed a single protein band with > 95% purity (Fig. 7A). The scFv bound in a dose-dependent manner to the soluble S1 (Fig. 7B) in ELISA, and the minimum detectable antigen S1 protein concentration was 6 ng/µL.

### Interaction between IgY-scFv and SARS-CoV-2 S1

**Fig. 8:**
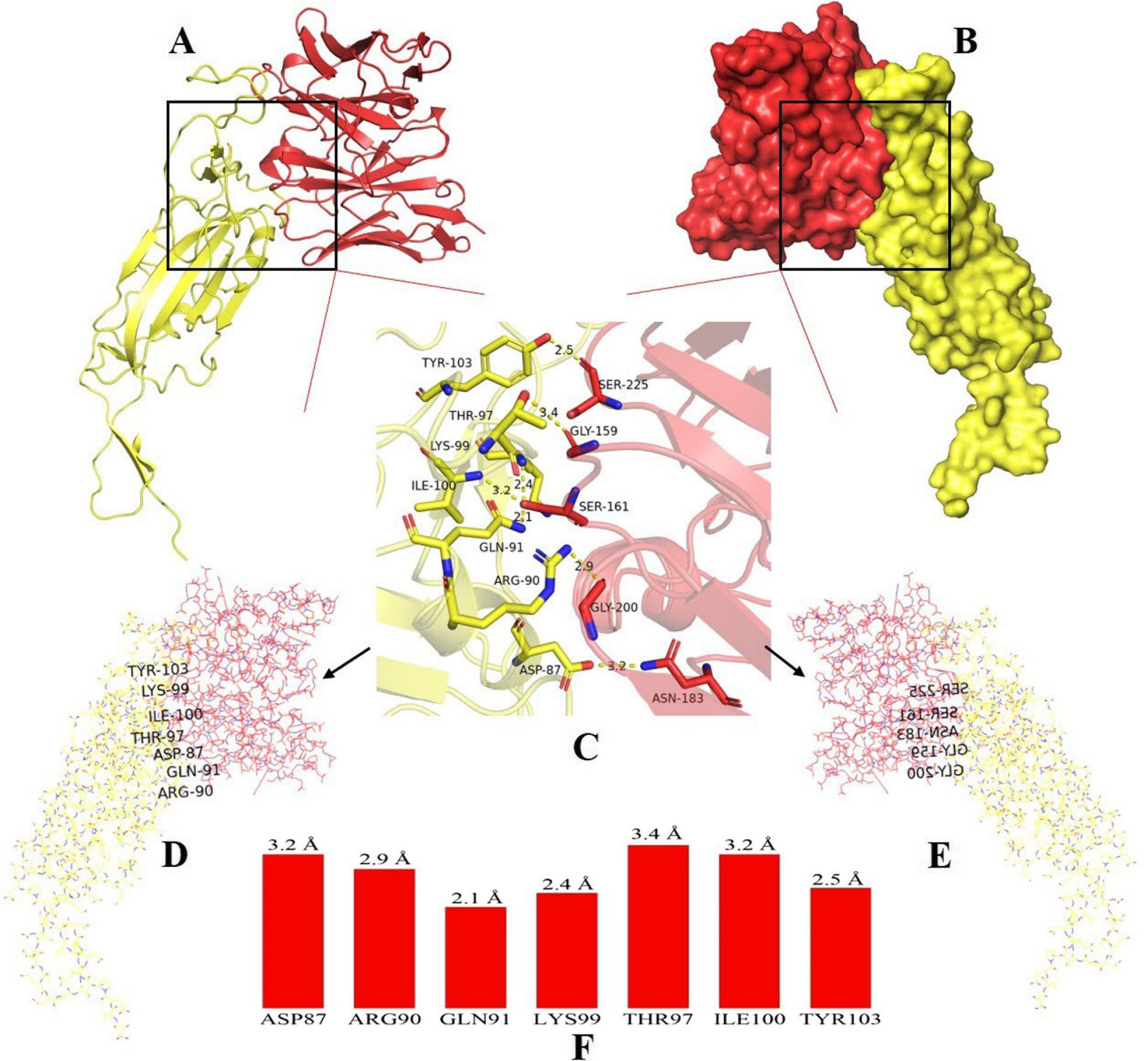
SARS-CoV-2 S1 protein-IgY scFv interaction and interface characterization. **A** the interacting interface residues in *yellow* and *red* cartoons. **B** the complex of scFv and RBD in surface view (*yellow* and *red*, respectively). **C**, **D** and** E** the interacting residues are shown: yellow (scFv) and red (RBD). **F** the interaction energetics quantification of hydrogen bond in scFv residues

ScFv-RBD docking identified the interacted hot-spot residues. The ASP87, ARG90, GLN91, THR97, LYS99, ILE100 and TYR103 of scFv contributed significantly to the formation of hydrogen bonds with residues GLY159, SER161, ASN183, GLY200 and SER225 of RBD, respectively (Fig. 8D). These epitopes are primarily located in the CDR3 domain of the IgY-scFv. Structure-based rational design of scFv with binding capacity to S1 protein may facilitate development of neutralizing antibodies (nAbs) for the suppression of viral infection.

## Discussion

The world has invested an unprecedented amount of resources to achieve a common goal of controlling and eradicating SARS-CoV-2. Specific antibodies are powerful tools for SARS-CoV-2 immunoassay analysis and providing passive immunization to tackling the infection (Casadevall and Pirofski 2020).

A series of anti-SARS-CoV-2 antibodies and vaccines have been being developed emergently. Several major anti-spike protein mammalian mAbs, S309/S2E12, 2B04/47D11, COV2-2130/COV2-2196, REGN10933/REGN10987 and LY-CoV555, were evaluated for their protective efficacy for Emergency Use Authorization (EUA). Limited data indicated that a combined therapy of several antibodies (S309/S2E12, COV2-2130/COV2-2196 and REGN10933/REGN10987) conferred better protection, as compared to the cases when the antibodies were applied solely (Chen et al. 2021; Gottlieb et al. 2021), this highlights the importance that we may need a toolbox of diversified antibodies and cocktail approach by targeting to distinct epitopes and mutants in order to achieve the best composite effect of therapy and prevention in the future. The VHH nanobodies from dromedary camels by phage display have high affinity for the RBD and broad neutralization activities against SARS-CoV-2, efficiently protects transgenic mice from the lethal challenge of virus (Hong et al. 2021). However, although the polyclonal human IgG (SAB-185) can protecte the human ACE2 transgenic Syrian hamster model from fatal disease and minimized clinical signs of infection, a control convalescent human serum sample was less effective at neutralizing the variant (Gilliland et al. 2021).

Here, we are providing alternative and novel solutions in generating avian sourced anti-SARS-CoV-2 polyclonal IgY, and more importantly, developing IgY-scFv antibodies by using *M13KO7* phage display technology, with the S1 protein as antigen.

As discussed recently, immunizing chickens with recombinant S1 protein can produce IgY neutralizing antibodies against the SARS-CoV-2 spike protein S1 subunit, and the production of large amounts of IgY that inhibits SARS-CoV-2 virus binding and replication is feasible for incorporation into an intranasal spray and/or other mucosal protection products may be effective at reducing infection and spread of COVID-19 (Artman et al. 2021; Bao et al. 2021). The large phylogenetic distance between mammals and birds allow IgY antibodies to recognize certain mammalian conserved protein epitopes (Hädge et al. 1980; Zhang et al. 2017; Pérez de la Lastra et al; 2020). The neutralized IgY antibodies, particularly the polyclonal IgY, as a potential passive immunization intervention against infections of emerging pathogens, including SARS-CoV-2, and safely used as a nasal, oral spray and gargle. In addition, IgY does not interact with mammalian Fc receptors or activate the mammalian complement system, so it can avoid triggering antibody-dependent enhancement (ADE) of disease and complement-mediated adverse inflammatory responses (Kovacs-Nolan and Mine 2012), therefore, it could be beneficial to administer neutralizing IgY (or, after genetic modification) intravascularly to treat SARS-CoV-2 infection (Lee et al. 2010; Lu et al. 2020). In this study, the chicken polyclonal IgY prepared can significantly inhibit the entry of SARS-CoV-2 virus into cells in vitro (Data not shown, Chinese Patent No. CN113402603A). The global effort to prevent SARS-CoV-2 infections will have to continue for some period, IgY antibodies from hen egg yolk could be a good alternative because of their viability for large-scale commercial production and the relative non-invasive methods used to prepare them.

In this study, the IgY-scFv bound to S1 protein efficiently and sensitively, and the minimum detectable antigen S1 protein concentration was 6 ng/µL (Fig. 7). The docking study suggested that the multiple interacted epitopes on the IgY-scFv (Fig. 8) were primarily located in the CDR3 domain. Our IgY-scFv as a parameter can be considered as the mode of humanization for better improvement (Nishibori et al. 2006), and various studies have been reported that the use of chimeric chicken IgY-scFv antibodies can be extended further to other mammalian species in human and veterinary applications (Tsurushita et al. 2004; Schusser et al. 2013).

This study is the first attempt to generate and characterize anti-SARS-CoV-2 avian highly specific IgY-scFv. Due to the restriction and difficulty in accessing SARS-CoV-2 virus and clinical samples, we were not able to further validate the obtained IgY-scFv in virus and animal models. However, as a pilot study, our work offers a possibility that the obtained IgY-scFv could be referenced as a parent structure for further antibody design in order to achieve better neutralization capacity to effectively block the SARS-CoV-2 infection. Furthermore, this IgY-scFv structure can be used for further engineering and optimization, which allows not only efficient mutation of antibody fragments with higher affinity to antigen, but also generation of functional antibody mimics/peptidomimetics for improved immunoassay and therapy (Baloch et al. 2016; Ge et al. 2020, 2021).

Anti-SARS-CoV-2 IgY-scFv and IgY antibodies were generated. The obtained IgY-scFv showed high binding sensitivity and binding capacity to the S1 protein, and the multiple epitopes on the IgY-scFv interact with multiple residues on SARS-CoV-2 spike protein RBD to form hydrogen bonds, these epitopes are primarily located in the CDR3 domain of the IgY-scFv. The IgY-scFv together with the polyclonal IgY we obtained, these antibodies could be applied for different biomedical scenarios in fighting against COVID-19 than mammalian antibodies.

## Data Availability

All data generated or analyzed during this study are included in this published article.

## Abbreviations

COVID-19: Coronavirus disease 2019
SARS-CoV-2: Severe acute respiratory syndrome coronavirus 2
ACE2: Angiotensin converting enzyme 2
RBD: Receptor binding domain
IgY-scFv: Chicken IgY single chain variable fragment antibody
S: Spike protein
nAbs: Neutralizing antibodies
IPTG: Isopropyl β-D-1thiogalactopyranoside
PBS: Phosphate buffered solution
RT-PCR: Real-time polymerase chain reaction
POCT: Point-of-care testing
mAb: Monoclonal antibody
VH: Heavy variable fragment
VL: Light chain variable fragment
RCF: Residues contact frequency
CDR3: Complementary determining region 3
pfu: Plaque forming units
EUA: Emergency Use Authorization
ADE: Antibody-dependent enhancement
